# Supplementary material for: P300 promotes tumor recurrence by regulating radiation-induced conversion of glioma stem cells to vascular-like cells
Source: Nat Commun. 2022 Oct 19;13:6202. doi: 10.1038/s41467-022-33943-0 (PMC9582000; doi:10.1038/s41467-022-33943-0)
Supplement: Supplementary file 1 — supplementary Information [file 41467_2022_33943_MOESM1_ESM.pdf]

**Supplementary Table 1:** Antibodies

| Antibodies | Source | Catalogue number | Dilution |
|---|---|---|---|
| Mouse monoclonal anti- CD31, JC70A, human | Agilent DAKO | M082329-2 | 1:200 |
| Mouse monoclonal anti- CD144/VE-CADHERIN, Clone BV9, human | Biolegend | 348502 | 1:100 |
| Mouse monoclonal anti- DESMIN, Clone D33 human | Agilent DAKO | M076001-2 | 1:100 |
| Mouse monoclonal anti- aSMA, Clone 1A4 human | Millipore Sigma | A2547 | 1:500 |
| Polyclonal Goat anti-VE-CADHERIN, human | R&D systems | AF938 | 1:100 |
| Mouse monoclonal anti-VE-CADHERIN, Clone BV9, human | Biolegend | 348502 | 1:100 |
| Rabbit polyclonal anti-GFP | Novus Biologicals | NB600-308 | 1:500 |
| Mouse monoclonal anti-mCherry | Novus Biologicals | NBP1-96752 | 1:500 |
| Chicken polyclonal anti-GFP | Novus Biologicals | NB100-1614 | 1:500 |
| Chicken polyclonal anti-mCherry | Millipore Sigma | AB356481 | 1:500 |
| CD31-PE (WM59), human | Biolegend | 303105 | 1:50 |
| CD144-APC (REA199), human | Miltenyi Biotec | 130-100-708 | 1:50 |
| CD146-APC, Clone P1H12, human | Biolegend | 361015 | 1:100 |
| CD248-647, Clone B1/35 human | BD Pharmingen | 564994 | 1:100 |
| CD133/2-PE, clone 293C3 human | Miltenyi Biotec | 130-113-186 | 1:50 |
| Rabbit monoclonal anti-B-actin | Cell Signaling Technology | 4970S | 1:5000 |
| Rabbit polyclonal anti-Histone 3 (D1H2) | Cell Signaling Technology | 4499 | 1:1000 |
| Rabbit polyclonal anti-AcH3 (K27) | Cell Signaling Technology | 4353 | 1:1000 |
| Rabbit polyclonal anti-GFAP | Agilent DAKO | GA52461-2 | 1:500 |
| Mouse monoclonal anti-NESTIN, Clone 10C2 human | Millipore Sigma | MAB5326 | 1:500 |
| Rabbit monoclonal anti-VIMENTIN (D21H3) | Cell Signaling Technology | 5741S | 1:500 |
| Rabbit monoclonal anti-N-CADHERIN (D4R1H) | Cell Signaling Technology | 13116S | 1:200 |
| Rabbit monoclonal anti-SOX2 (D9B8N) | Cell Signaling Technology | 23064S | 1:50 |
| Rabbit monoclonal anti-P300 (D8Z4E) | Cell Signaling Technology | 86377S | 1:100 |
| Rabbit monoclonal, anti-DESMIN (D93F5) | Cell Signaling Technology | 5332S | 1:200 |
| Rabbit monoclonal, anti-VE-CADHERIN | Cell Signaling Technology | 2500S | 1:100 |
| Goat polyclonal anti-VE-CADHERIN, mouse | R&D Systems | AF1002 | 1:100 |
| Tomato Lectin-DyLight 649 | Vector Laboratories | DL-1178-1 | 1:1000 |

| | | | |
|---|---|---|---|
| Goat anti-Mouse IgG1, Alexa Fluor 568 | Thermo Fisher | A21124 | 1:250 |
| Goat anti-Mouse IgG2a, Alexa Fluor 548 | Thermo Fisher | A21134 | 1:250 |
| Goat anti-rabbit Alexa Fluor 488 | Abcam | Ab150077 | 1:500 |
| Donkey anti-mouse IgG, Alexa Fluor 568 | Fisher Scientific | A10037 | 1:200 |
| Donkey anti-goat Alexa Fluor 488 | Abcam | Ab150129 | 1:500 |
| Goat anti-rabbit Alexa Fluor 568 | Invitrogen | A11011 | 1:250 |
| Goat anti-chicken IgY, Alexa Fluor 488 | Fisher Scientific | A11039 | 1:500 |

**Supplementary Table 2:** Primers for quantitative RT-PCR

| Genes | Forward primer | Reverse primer |
|---|---|---|
| **Human Oligonucleotides** | | |
| 18srRNA | GGCCCTGTAATTGGAATGAGTC | CCAAGATCCAACTACGAGCTT |
| PECAM1 | CCAAGGTGGGATCGTGAGG | TCGGAAGGATAAAACGCGGTC |
| CDH5 | AAGCGTGAGTCGCAAGAATG | TCTCCAGGTTTTCGCCAGTG |
| FLT1 | GAAAACGCATAATCTGGGACAGT | GCGTGGTGTGCTTATTTGGA |
| VWF | CCGATGCAGCCTTTTCGGA | TCCCCAAGATACACGGAGAGG |
| ENG | CGCCAACCACAACATGCAG | GCTCCACGAAGGATGCCAC |
| EDN1 | AAGGCAACAGACCGTGAAAAT | CGACCTGGTTTGTCTTAGGTG |
| DES | GAGACCATCGCGGCTAAGAAC | GTGTAGGACTGGATCTGGTGT |
| ACTA2 | CTATGAGGGCTATGCCTTGCC | GCTCAGCAGTAGTAACGAAGGA |
| PDGFRB | AGCACCTTCGTTCTGACCTG | TATTCTCCCGTGTCTAGCCCA |
| MCAM | AGCTCCGCGTCTACAAAGC | CTACACAGGTAGCGACCTCC |
| CD248 | TGGTGCCAACGTGTGTCTTTT | AGCGATAGCAGTCAGTGATGC |
| ANGPT1 | AGCGCCGAAGTCCAGAAAAC | TACTCTCACGACAGTTGCCAT |
| SOX2 | GCCGAGTGGAAACTTTTGTCG | GGCAGCGTGTACTTATCCTTCT |
| NES | CTGCTACCCTTGAGACACCTG | GGGCTCTGATCTCTGCATCTAC |
| GFAP | CTGCGGCTCGATCAACTCA | TCCAGCGACTCAATCTTCCTC |
| POU5F1 | GGGAGATTGATAACTGGTGTGTT | GTGTATATCCCAGGGTGATCCTC |
| NANOG | TTTGTGGGCCTGAAGAAAACT | AGGGCTGTCCTGAATAAGCAG |

| | | |
|---|---|---|
| KLF4 | CGGACATCAACGACGTGAG | GACGCCTTCAGCACGAACT |
| CD15 | GATCTGCGCGTGTTGGACTA | GAGGGCGACTCGAAGTTCAT |
| CDH1 | AAAGGCCCATTTCCTAAAAACCT | TGCGTTCTCTATCCAGAGGCT |
| CDH2 | AGCCAACCTTAACTGAGGAGT | GGCAAGTTGATTGGAGGGATG |
| SNAI1 | TCGGAAGCCTAACTACAGCGA | AGATGAGCATTGGCAGCGAG |
| SNAI2 | TGTGACAAGGAATATGTGAGCC | TGAGCCCTCAGATTTGACCTG |
| TWIST1 | GTCCGCAGTCTTACGAGGAG | GCTTGAGGGTCTGAATCTTGCT |
| BMI1 | GCTGCCAATGGCTCTAATGAA | TGCTGGGCATCGTAAGTATCTT |
| **Mouse Oligonucleotides** | | |
| *18srRNA* | GTAACCCGTTGAACCCCATT | CCATCCAATCGGTAGTAGCG |
| *Pecam1* | CTGCCAGTCCGAAAATGGAAC | CTTCATCCACCGGGGCTATC |
| *Cdh5* | CACTGCTTTGGGAGCCTTC | GGGGCAGCGATTCATTTTTCT |
| *Eng* | CCCTCTGCCCATTACCCTG | GTAAACGTCACCTCACCCCTT |
| *Edn1* | GCACCGGAGCTGAGAATGG | GTGGCAGAAGTAGACACACTC |
| *Des* | GTGGATGCAGCCACTCTAGC | TTAGCCGCGATGGTCTCATAC |
| *Mcam* | CCCAAACTGGTGTGCGTCTT | GGAAAATCAGTATCTGCCTCTCC |
| *Cd248* | CAACGGGCTGCTATGGATTG | GCAGAGGTAGCCATCGACAG |
| *Pdgfrb* | CAAGAAGCGGCCATGAATCAG | CGGCCCTAGTGAGTTGTTGT |
| *Sox2* | GCGGAGTGGAAACTTTTGTCC | CGGGAAGCGTGTACTTATCCTT |
| *Pou5f1* | GGCTTCAGACTTCGCCTCC | AACCTGAGGTCCACAGTATGC |
| *Nanog* | TCTTCCTGGTCCCCACAGTTT | GCAAGAATAGTTCTCGGGATGAA |
| *Klf4* | GTGCCCCGACTAACCGTTG | GTCGTTGAACTCCTCGGTCT |
| *Nes* | CCCTGAAGTCGAGGAGCTG | CTGCTGCACCTCTAAGCGA |
| *Gfap* | CCCTGGCTCGTGTGGATTT | GACCGATACCACTCCTCTGTC |

**Supplementary Fig.1 Single-cell sequencing shows diverse functional states of glioma cells, related to Fig.1**

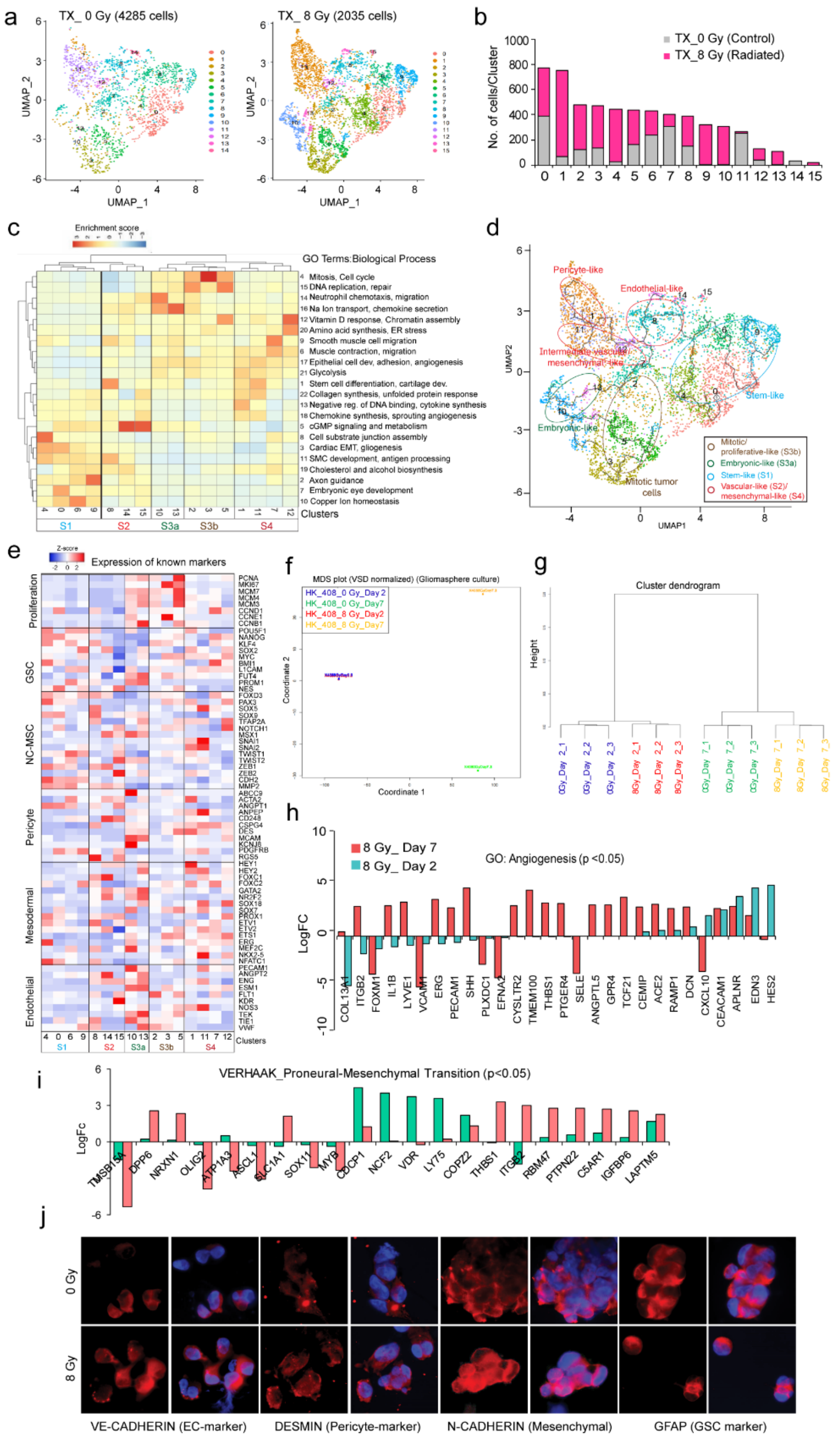


a. UMAP plot of glioma cells from control (0 Gy) and radiated (8 Gy) tumor xenografts (TX).

b. Histogram shows the number of cells per cluster in each group.

c. Heatmap of differential expression of co-expressed gene modules. The subgroups are highlighted in black boxes.

d. UMAP plot of pseudotime trajectory analysis of all the clusters. The functional states representing the subgroups (circled in color) are shown in the inset.

e. Heatmap shows average expression of markers in each cluster from tumor xenografts.

f. g. MDS plot and cluster dendrogram of gene expression differences between control and day 2 and day 7-radiated gliomaspheres.

h. Graph shows LogFc expression of genes in the GO: Angiogenesis category in radiated vs control glioma cells.

i. LogFC values of genes associated with VERHAAK_Glioblastoma_Mesenchymal gene set in control and radiated gliomaspheres.

j. Immunostaining of endothelial (VE-CADHERIN), pericyte (DESMIN), GSC (GFAP) and mesenchymal marker (VIMENTIN) in control and radiated gliomaspheres. Scale bars, 50um.

**Supplementary Fig. 2 Radiation promotes endothelial and pericyte marker expression, related to Fig. 2**

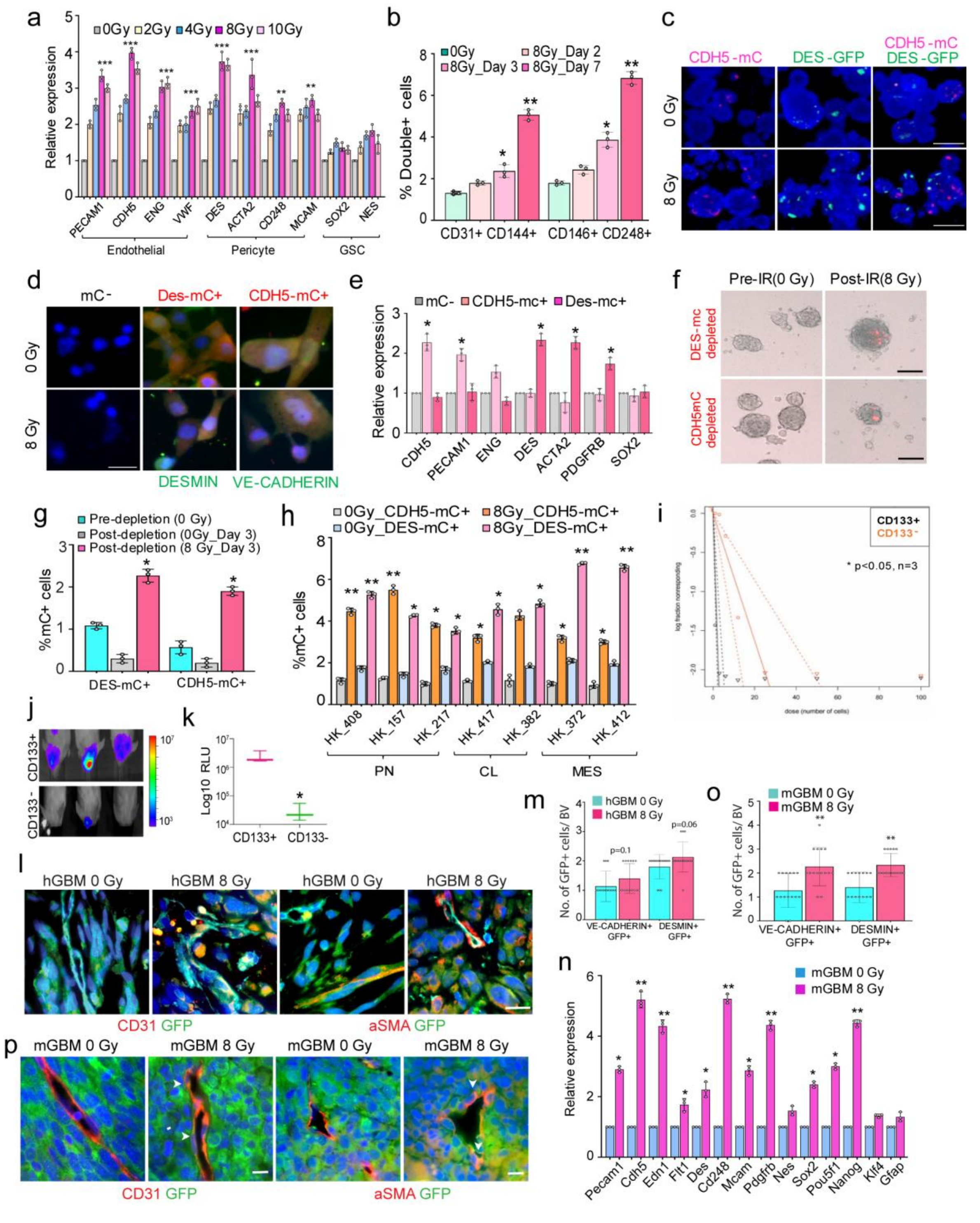

a. Relative expression of endothelial, pericyte and GSC genes in control, low and high-dose radiated cells. Error bars represent mean ± SD. N=3 biological replicates. *** and ** indicates $p<0.0005$ and $p<0.005$, one-way ANOVA, post-hoc t-test.

b. Flow cytometric quantitation of endothelial (CD31+ CD144+) and pericyte (CD146+ CD248+) markers in control and 2, 3 and 7 day-radiated glioma cells. N=3 biological replicates. * and ** indicates $p<0.05$ and $p<0.005$, one-way ANOVA.

c. mCherry expression in control and radiated gliomaspheres. Scale bars, 150µm.

d. Immunostaining of VE-CADHERIN and DESMIN in FACS sorted tumor (mC-) and transdifferentiated cells (CDH5-mC+/DES-mC+). Scale bars, 50µm.

e. Relative expression of endothelial, pericyte and GSC markers in sorted tumor (mC-) and transdifferentiated cells (CDH5-mC+ and DES-mC+) in control and radiated gliomaspheres. N=3 biological replicates. ** and * indicates $p<0.005$ and $p<0.05$, one-way ANOVA.

f. g. mCherry expression in sorted, reporter-depleted control and radiated cells. Scale bars, 200µm. Graph shows quantitation of mCherry+ cells in each group. N=3 biological replicates. * indicates $p<0.05$, one-way ANOVA.

h. Flow cytometric quantitation of percentage of mCherry+ cells in endothelial and pericyte-reporter transduced control and radiated gliomaspheres from multiple patient-derived lines. PN-proneural, CL- classical and MES- Mesenchymal subtypes. N=3 biological replicates. * and ** indicates $p<0.05$, and $p<0.005$, unpaired two-tailed t-test.

i. Limiting dilution analysis of stem cell frequency in CD133+ and CD133- fractions. Chi-square goodness of fit-test. N=3 independent experiments.

j. k. Tumor growth in CD133+ and CD133- cells transplanted in mice. Graph shows quantitation of luminescence between the groups. N=3 mice per group, * indicates $p<0.05$, two-tailed t-test.

l. Immunostaining of CD31 and aSMA with GFP in tumor cells from control and radiated xenografts. Scale bars, 25um.

m. Quantitation of marker+ GFP+ hGBM tumor cells in blood vessels (BV) per section. Data is represented as mean ± SD. N=3 mice per group, ** indicates p<0.005, unpaired two-tailed t-test.

n. Relative expression of endothelial, pericyte and GSC markers in control and radiated mGBM cells. N=3 biological replicates. * and ** indicates p<0.05, unpaired two-tailed t-test.

o. Quantitation of marker+ GFP+ mGBM tumor cells in blood vessels (BV) per tumor section. Data is represented as mean ± SD. N=3 mice per group, ** indicates p<0.005, unpaired two-tailed t-test

p. Immunostaining of CD31 and aSMA with GFP in tumor cells from control and radiated murine GBM tumors. Scale bars, 25um.

**Supplementary Fig. 3 iGEC show characteristics of normal vascular endothelial cells, related to Fig. 3**

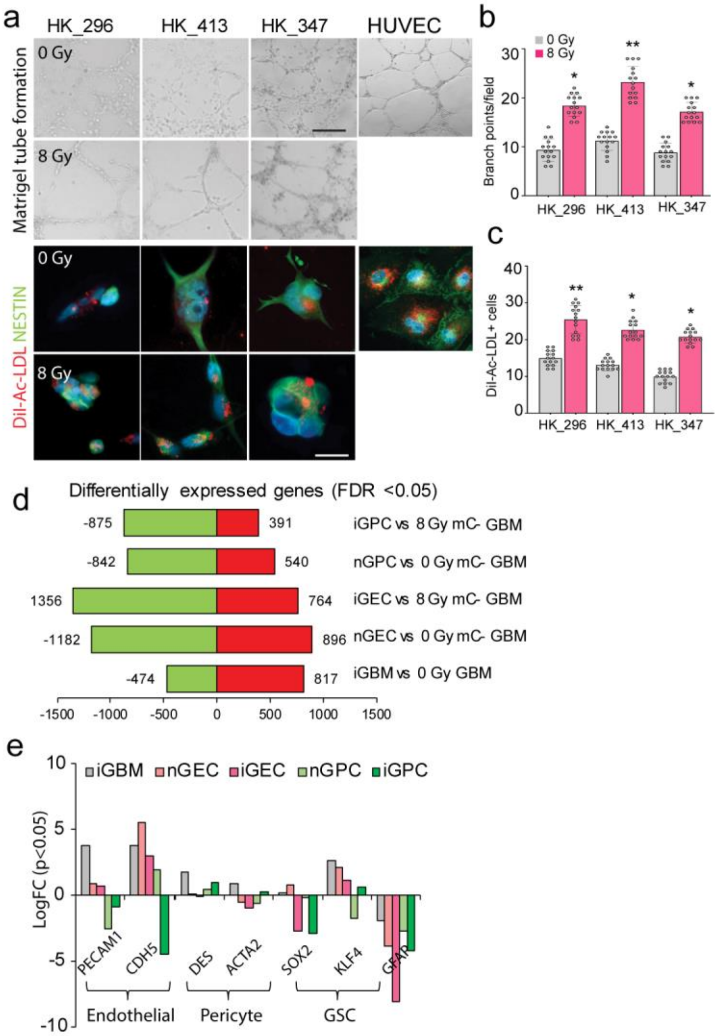


a-c. Tubular network formation on matrigel and immunostaining of NESTIN and Di-AcLDL uptake of 7-day cultured control and radiated glioma cells. HUVEC were used as positive control. Scale bars, 100 and 50µm. Quantitation of branch points and DiI-Ac-LDL+ cells per field is shown in the graph. N=3 biological replicates. * and ** indicates $p<0.05$, and $p<0.005$, unpaired two-tailed t-test

d. Graph shows the differentially expressed genes between GBM and transdifferentiated cells.

e. Graph shows LogFC expression values of endothelial, pericyte and GSC markers in the different fractions.

**Supplementary Fig.4 Depletion of iGEC and iGPC diminishes tumor growth post-radiation treatment, related to Fig. 4**

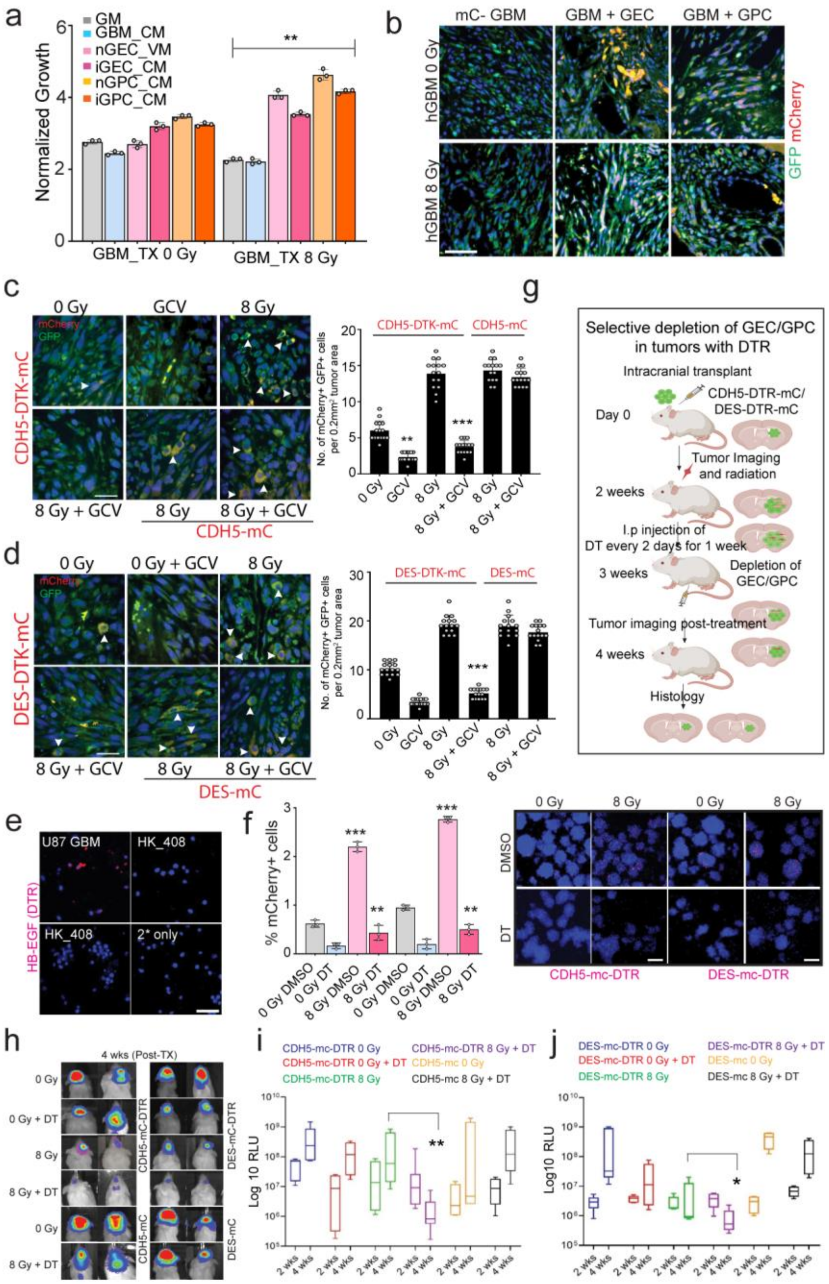


a. Proliferation of sorted tumor cells from xenografts in GEC and GPC conditioned media. Error bars represent mean±SD, N=3 biological replicates, ** p<0.005, one-way ANOVA

b. Immunostaining of GFP and mCherry in control and radiated tumors co-transplanted with GEC/GPC. Scale bars, 200µm.

c. d. Immunostaining of GFP and mCherry in control and radiated tumors treated with Ganciclovir (GCV) to deplete GEC/GPC. Quantitation of number of GFP+ mCherry+ cells in each group. N=5 mice per group, * and ** indicates p-value< 0.05 and <0.005, one-way ANOVA, post-hoc t-test

e. Immunostaining of HB-EGF (DTR, red) in U87 GBM and HK_408 cells

f. Quantitation of mCherry+ cells pre-and post-radiation and diphtheria toxin (DT) treatment. Images show expression of mCherry in control and radiated cells after DMSO or DT treatment. Error bars represent mean ± SD, N=3 mice per group, ** and *** indicates p<0.005 and p<0.0005 unpaired two-tailed t-test.

g. Schematic outlines the depletion of GEC/GPC using DTR-DT strategy in tumor xenografts

h-j. Images of mice showing tumor growth post-treatment with DT. Box plots show quantitation of tumor growth by luminescence pre- (2 week) and post-radiation and DT treatment (4 weeks). Box plots display the median, $25^{th}$ and $75^{th}$ percentile, and whiskers extend from min to max value. N=5 mice per group, * and ** indicates p<0.05 and p<0.005, one-way ANOVA and post-hoc t-test.

**Supplementary Fig. 5 Radiation alters chromatin accessibility in vascular gene regions, related to Fig. 5**

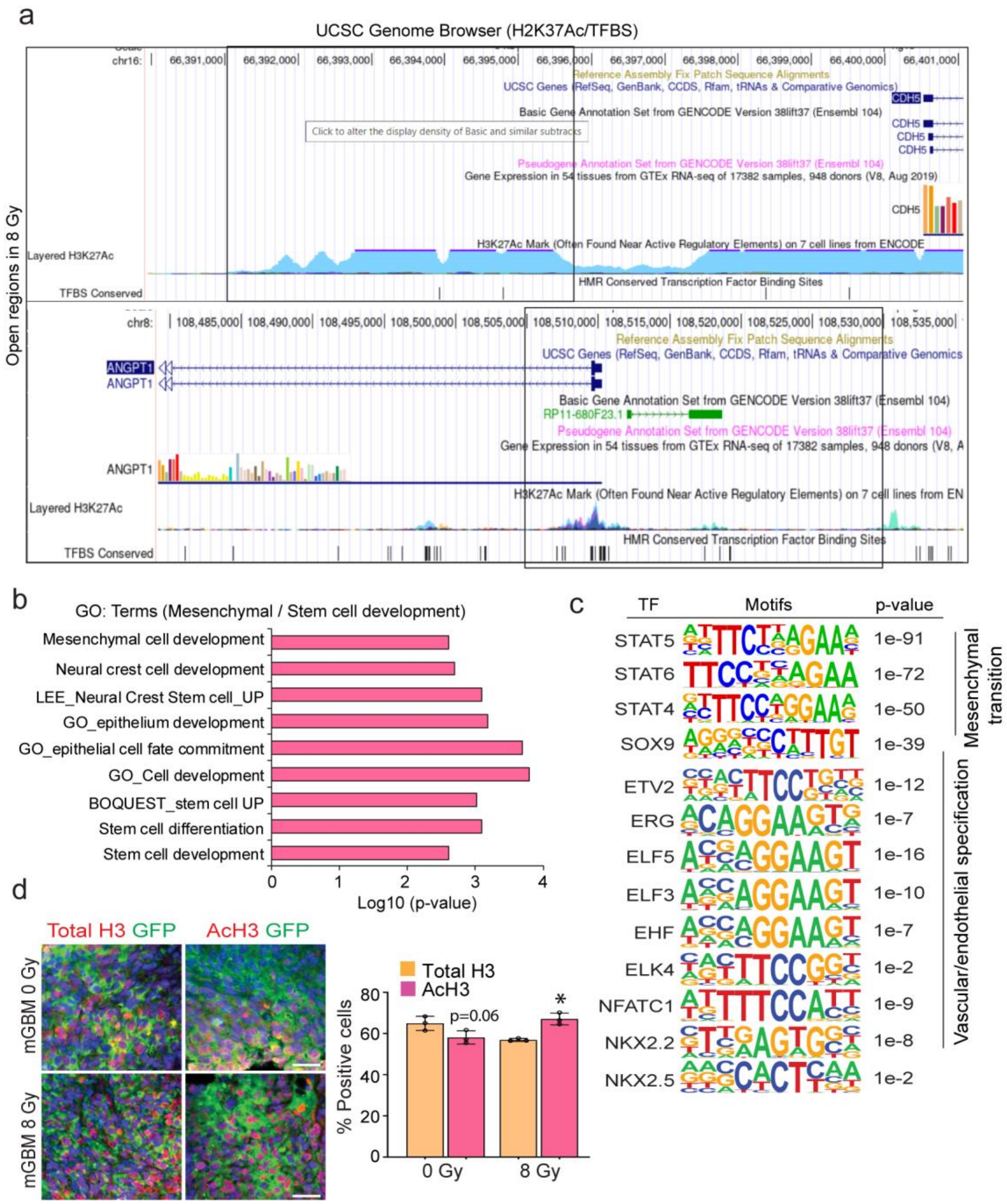


a. UCSC Genome Browser view of the CDH5 and ANGPT1 gene regions (hg19 assembly) with known H3K27Ac mark and TFBS sites. Region highlighted in black box is differentially open in radiated glioma cells.

b. Graph shows significantly enriched gene ontology (GO) terms related to mesenchymal and stem cell development associated with genes differentially open in radiated gliomaspheres.

c. Motifs significantly enriched in differentially open regions in radiated gliomaspheres. p-values are derived using hypergeometric or binomial distribution in HOMER.

d. Immunostaining of AcH3 and total H3 (red) in tumor cells (GFP, green) in control and radiated mouse GBM tumors. Quantitation of percentage of positive cells per section. N=3 mice per group, * indicates $p<0.05$, unpaired two-tailed t-test. Scale bars, 100μm.

**Supplementary Fig. 6 Blocking P300 HAT activity inhibits radiation-induced vascular-like conversion, related to Fig. 6**

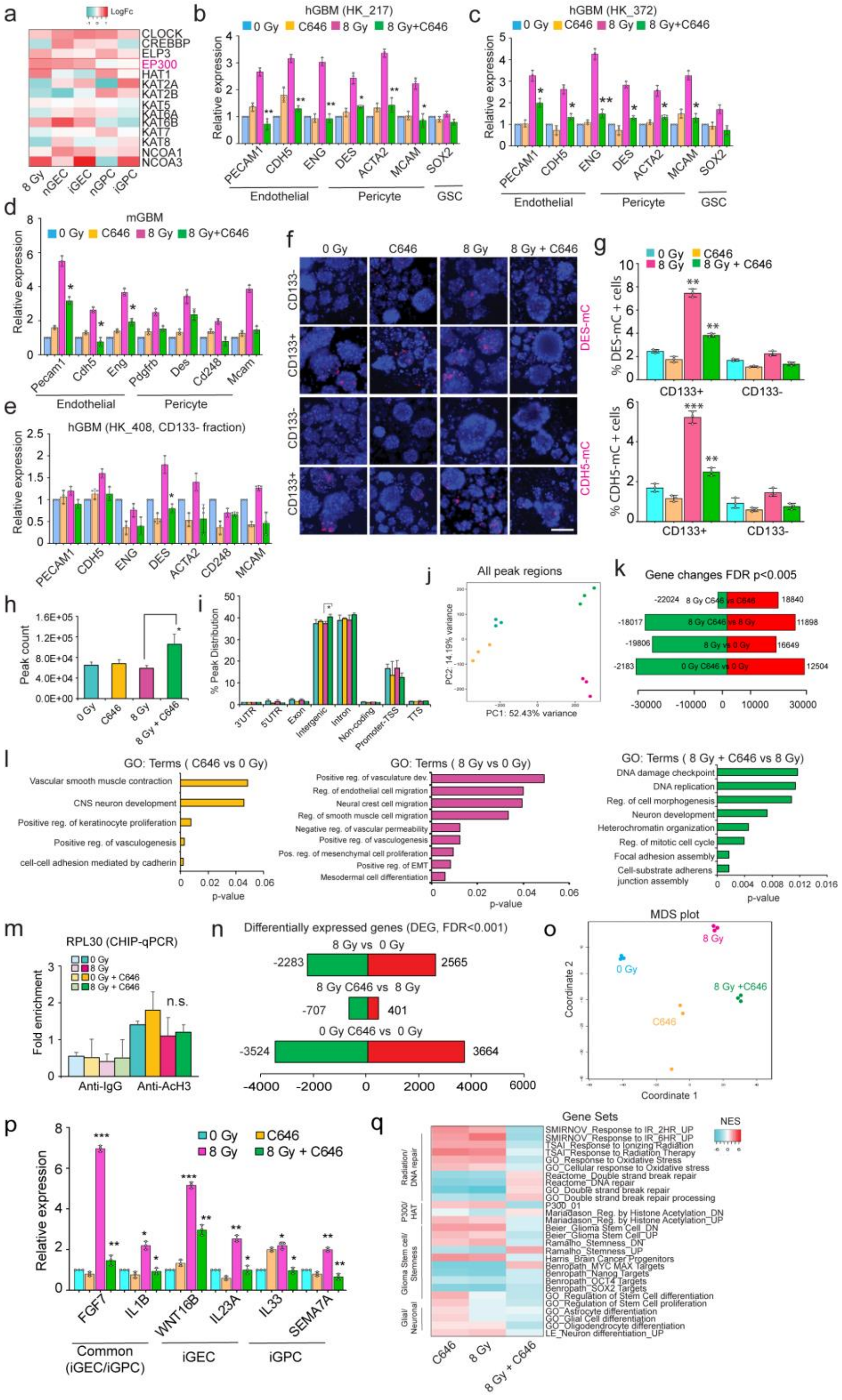

a. Heatmap shows LogFC expression of HAT transcripts from RNA-sequencing of sorted tumor (8 Gy GBM) and transdifferentiated cells (nGEC/iGEC and nGPC/iGPC).

b-e. Relative expression of endothelial, pericyte markers in control and radiated hGBM lines, mGBM, and sorted CD133- hGBM fractions treated with C646. Error bars represent mean ± SD, N=3 biological replicates, ** and * indicates $p<0.005$ and $p<0.05$, unpaired two-tailed t-test.

f. g. mCherry+ expression in sorted CD133+ and CD133- fractions after radiation and C646 treatment. Quantitation of percentage of endothelial (CDH5-mC) and pericyte (DES-mC) reporter expression in each group. N=3 biological replicates, ** and *** indicates $p<0.005$ and $p<0.0005$, one-way ANOVA and post-hoc t-test.

h. i. Average peak counts and peak distribution across genomic regions from ATAC-sequencing of control (0 Gy), HAT inhibitor (C646), radiated (8 Gy) and combined radiation + HAT inhibitor (8 Gy+ C646) treated gliomaspheres. N=3 biological replicates, * $p<0.05$, one-way ANOVA.

j. PCA plot of all peak regions from control (0 Gy), HAT inhibitor (C646), radiated (8 Gy) and combined radiation + HAT inhibitor (8 Gy+ C646) treated gliomaspheres.

k. Genes changes associated with peaks between control, radiated and C646 treated groups

l. GO_terms associated with differentially open regions in C646 treated and radiated cells.

m. Fold enrichment of H3K27Ac in RPL30 gene in different groups. N=3 biological replicates, and n.s. indicates not significant, one-way ANOVA.

n. Differentially gene expression between control, radiated and C646 treated groups.

o. MDS plot of gene expression in control, radiated and C646 treated gliomaspheres.

p. Relative expression of iGEC and iGPC-enriched factors in control and radiated gliomaspheres treated with or without C646. N=3 biological replicates, ***, ** and * indicates $p<0.0005$, $p<0.005$ and $p<0.05$, one-way ANOVA and post-hoc t-test.

q. Heatmap shows NES (normalized enrichment scores, $p<0.05$) of gene sets in control and radiated gliomaspheres with or without C646 treatment.

**Supplementary Fig. 7 EP300-deficient glioma cells show reduced H3K27Ac and vascular marker expression post-radiation, related to Fig. 7**

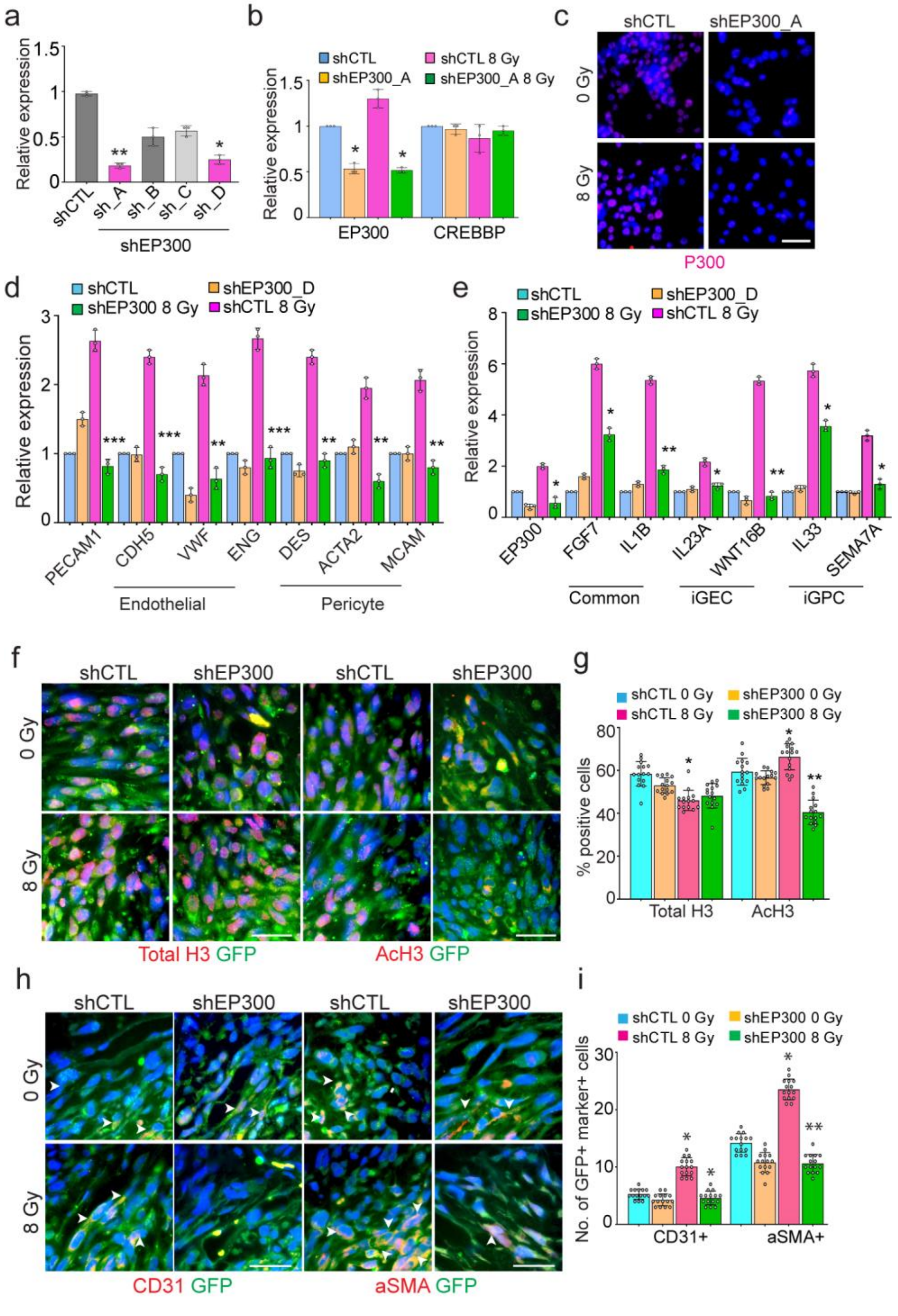

a. Relative expression of EP300 in control (shRNA-scrambled) and knockdown (shRNAs-EP300-A and D) cells in gliomaspheres. N=3 biological replicates, * and ** indicates $p<0.05$ and $p<0.005$, unpaired two-tailed t-test.

b. Relative expression of EP300 and CREBBP in non-radiated and radiated control and EP300-knockdown (shEP300_A) glioma cells. N=3 biological replicates, * $p<0.05$, one-way ANOVA.

c. Immunostaining of P300 in control and knockdown (shEP300_A) cells.

d. Relative expression of endothelial and pericyte markers in non-radiated and radiated control and EP300-knockdown (shEP300_A and shEP300_D) glioma cells. N=3 biological replicates, *** and ** indicates $p<0.0005$ and $p<0.005$, one-way ANOVA.

e. Relative expression of iGEC and iGPC factors in non-radiated and radiated control and EP300-knockdown (shEP300_A and shEP300_D) glioma cells. N=3 biological replicates, ** and * indicates $p<0.005$ and $p<0.05$, one-way ANOVA.

f. g. Immunostaining of Total H3 and AcH3, and GFP in control and knockdown tumor cells in control and radiated xenografts. Scale bars, 50µm Graph shows quantitation of marker+ GFP+ positive in each group. N=3 mice per group, * and ** indicates $p<0.05$, $p<0.005$, one-way ANOVA.

h. i. Immunostaining of CD31 and aSMA and GFP in tumor sections. Scale bars, 50µm. Quantitation of GFP+ marker+ cells in tumor mass is shown in the graph. N=3 mice per group, * indicates $p<0.05$ and ** $p<0.005$, one-way ANOVA.
