# Supplementary material for: P300 promotes tumor recurrence by regulating radiation-induced conversion of glioma stem cells to vascular-like cells
Source: Nat Commun. 2022 Oct 19;13:6202. doi: 10.1038/s41467-022-33943-0 (PMC9582000; doi:10.1038/s41467-022-33943-0)
Supplement: Supplementary file 3 — Reporting Summary [file 41467_2022_33943_MOESM3_ESM.pdf]

nature portfolio

Corresponding author(s): Harley Kornblum
Last updated by author(s): 9/27/2022

Reporting Summary

Nature Portfolio wishes to improve the reproducibility of the work that we publish. This form provides structure for consistency and transparency in reporting. For further information on Nature Portfolio policies, see our Editorial Policies and the Editorial Policy Checklist.

Statistics

For all statistical analyses, confirm that the following items are present in the figure legend, table legend, main text, or Methods section.

n/a	Confirmed	
☐	☒	The exact sample size (*n*) for each experimental group/condition, given as a discrete number and unit of measurement
☐	☒	A statement on whether measurements were taken from distinct samples or whether the same sample was measured repeatedly
☐	☒	The statistical test(s) used AND whether they are one- or two-sided<br>*Only common tests should be described solely by name; describe more complex techniques in the Methods section.*
☒	☐	A description of all covariates tested
☐	☒	A description of any assumptions or corrections, such as tests of normality and adjustment for multiple comparisons
☐	☒	A full description of the statistical parameters including central tendency (e.g. means) or other basic estimates (e.g. regression coefficient) AND variation (e.g. standard deviation) or associated estimates of uncertainty (e.g. confidence intervals)
☐	☒	For null hypothesis testing, the test statistic (e.g. *F*, *t*, *r*) with confidence intervals, effect sizes, degrees of freedom and *P* value noted<br>*Give P values as exact values whenever suitable.*
☒	☐	For Bayesian analysis, information on the choice of priors and Markov chain Monte Carlo settings
☒	☐	For hierarchical and complex designs, identification of the appropriate level for tests and full reporting of outcomes
☒	☐	Estimates of effect sizes (e.g. Cohen's *d*, Pearson's *r*), indicating how they were calculated

Our web collection on statistics for biologists contains articles on many of the points above.

Software and code

Policy information about availability of computer code

Data collection	Immunofluorescence Images were obtained using EVOS FL Auto Imaging System and LEICA LASX microscope application Suite X.
Data analysis	R-Package V.3.2.5, Cell Ranger v.3.0.2, Seurat v.3.1.1. were used for analysis of single-cell RNA sequencing data analysis. DEseq, Edge R was used for bulk RNA-sequencing analysis. For GSEA analysis, mSigDB ver 7.0 was used as reference. Peak calling for ATAC-sequencing was performed using MACS2, and HOMER was used for MOTIF enrichment and GO analysis was done using EnrichR. UCSC Browser and Integrated Genome Viewer were used to visualize the peaks from ATAC-sequencing. GraphPad Prism 8 was used for statistical analysis of all other data as described in the methods. FlowJo v10.8 was used to analyze cytometric data.

For manuscripts utilizing custom algorithms or software that are central to the research but not yet described in published literature, software must be made available to editors and reviewers. We strongly encourage code deposition in a community repository (e.g. GitHub). See the Nature Portfolio guidelines for submitting code & software for further information.

Data

Policy information about availability of data

All manuscripts must include a data availability statement. This statement should provide the following information, where applicable:

- Accession codes, unique identifiers, or web links for publicly available datasets
- A description of any restrictions on data availability
- For clinical datasets or third party data, please ensure that the statement adheres to our policy

All sequencing data has been submitted to Gene Expression Omnibus, and are available with the Accession number GSE207808. All other quantitative data from this

study can be obtained in the source data file provided with this paper or made available upon request to the corresponding author

Human research participants

Policy information about studies involving human research participants and Sex and Gender in Research.

Reporting on sex and gender	N/A
Population characteristics	N/A
Recruitment	N/A
Ethics oversight	All de-identified patient tissue samples were collected under informed consent and approved by UCLA Medical Institutional Review Board

Note that full information on the approval of the study protocol must also be provided in the manuscript.

Field-specific reporting

Please select the one below that is the best fit for your research. If you are not sure, read the appropriate sections before making your selection.

☒ Life sciences ☐ Behavioural & social sciences ☐ Ecological, evolutionary & environmental sciences

For a reference copy of the document with all sections, see nature.com/documents/nr-reporting-summary-flat.pdf

Life sciences study design

All studies must disclose on these points even when the disclosure is negative.

Sample size	Sampe size was determined on the basis of prior studies published by our laboratory and by the effect size between groups.
Data exclusions	No data was excluded from the analyses
Replication	To ensure reproducibility and rigor, all experiments were repeated a minimum of 2-3 independent times with atleast 3 to 5 replicates per condition with successful replication of the data
Randomization	For animal experiments, both male and female mice with tumors were randomized for radiation and drug treatments. For in vitro experiments, we chose cell lines that we previously tested and found to have tumor-formation capacity in mice, and then replicated the results in multiple other patient-derived lines.
Blinding	Blinding was done for experiments that required cell counting on immunostained tumor sections between control and treatment groups. For all single-cell sequencing experiments, data was analyzed in an integrated manner without sample IDs. For ATAC-sequencing experiments, samples were processed and analyzed in a blinded fashion by the bioinformatician. For in vitro culture experiments, blinding is not relevant as we were comparing radiated to non-radiated cells with reporters easy to distinguish the effects.

Reporting for specific materials, systems and methods

We require information from authors about some types of materials, experimental systems and methods used in many studies. Here, indicate whether each material, system or method listed is relevant to your study. If you are not sure if a list item applies to your research, read the appropriate section before selecting a response.

Materials & experimental systems

n/a	Involved in the study	
☐	☒	Antibodies
☐	☒	Eukaryotic cell lines
☒	☐	Palaeontology and archaeology
☐	☒	Animals and other organisms
☒	☐	Clinical data
☒	☐	Dual use research of concern

Methods

n/a	Involved in the study	
☒	☐	ChIP-seq
☐	☒	Flow cytometry
☒	☐	MRI-based neuroimaging

Antibodies

Antibodies used	Mouse monoclonal anti- CD31, JC70A, human Agilent DAKO M082329-2 1:200<br>Mouse monoclonal anti- CD144/VE-CADHERIN, Clone BV9, human Biolegend 348502 1:100<br>Mouse monoclonal anti- DESMIN, Clone D33 human Agilent DAKO M076001-2 1:100<br>Mouse monoclonal anti- aSMA, Clone 1A4 human Millipore Sigma A2547 1:500<br>Polyclonal Goat anti-VE-CADHERIN, human R&D systems AF938 1:100<br>Mouse monoclonal anti-VE-CADHERIN, Clone BV9, human Biolegend 348502 1:100<br>Rabbit polyclonal anti-GFP Novus Biologicals NB600-308 1:500<br>Mouse monoclonal anti-mCherry Novus Biologicals NBP1-96752 1:500<br>Chicken polyclonal anti-GFP Novus Biologicals NB100-1614 1:500<br>Chicken polyclonal anti-mCherry Millipore Sigma AB356481 1:500<br>CD31-PE (WM59), human Biolegend 303105 1:50<br>CD144-APC (REA199), human Miltenyi Biotec 130-100-708 1:50<br>CD146-APC, Clone P1H12, human Biolegend 361015 1:100<br>CD248-647, Clone B1/35 human BD Pharmingen 564994 1:100<br>CD133/2-PE, clone 293C3 human Miltenyi Biotec 130-113-186 1:50<br>Rabbit monoclonal anti-B-actin Cell Signaling Technology 4970S 1:5000<br>Rabbit polyclonal anti-Histone 3 (D1H2) Cell Signaling Technology 4499 1:1000<br>Rabbit polyclonal anti-AcH3 (K27) Cell Signaling Technology 4353 1:1000<br>Rabbit polyclonal anti-GFAP Agilent DAKO GA52461-2 1:500<br>Mouse monoclonal anti-NESTIN, Clone 10C2 human Millipore Sigma MAB5326 1:500<br>Rabbit monoclonal anti-VIMENTIN (D21H3) Cell Signaling Technology 5741S 1:500<br>Rabbit monoclonal anti-N-CADHERIN (D4R1H) Cell Signaling Technology 13116S 1:200<br>Rabbit monoclonal anti-SOX2 (D9B8N) Cell Signaling Technology 23064S 1:50<br>Rabbit monoclonal anti-P300 (D8Z4E) Cell Signaling Technology 86377S 1:100<br>Rabbit monoclonal, anti-DESMIN (D93F5) Cell Signaling Technology 5332S 1:200<br>Rabbit monoclonal, anti-VE-CADHERIN Cell Signaling Technology 2500S 1:100<br>Goat polyclonal anti-VE-CADHERIN, mouse R&D Systems AF1002 1:100<br>Tomato Lectin-DyLight 649 Vector Laboratories DL-1178-1 1:1000<br>Goat anti-Mouse IgG1, Alexa Fluor 568 Thermo Fisher A21124 1:250<br>Goat anti-Mouse IgG2a, Alexa Fluor 548 Thermo Fisher A21134 1:250<br>Goat anti-rabbit Alexa Fluor 488 Abcam Ab150077 1:500<br>Donkey anti-mouse IgG,Alexa Fluor 568 Fisher Scientific A10037 1:200<br>Donkey anti-goat Alexa Fluor 488 Abcam Ab150129 1:500<br>Goat anti-rabbit Alexa Fluor 568 In vitrogen A11011 1:250<br>Goat anti-chicken IgY, Alexa Fluor 488 Fisher Scientific A11039 1:500
Validation	Several antibodies used in this study have been previously validated by the manufacturer, or in prior publications, or validated by our laboratory (P300 antibody) on knockdown cells, or on cell types known to express them such as HUVEC (VE-CADHERIN, CD31). GFP and mCherry antibodies were validated by staining cells transduced with lentiviral reporters expressing GFP or mCherry. All antibodies used for human samples were tested in human lines, and antibodies for mouse samples were tested in mouse tumor lines. Manufacturer has information regarding species validation and is listed in the supplement table for each antibody with their clone number and catalog number.

Eukaryotic cell lines

Policy information about cell lines and Sex and Gender in Research

Cell line source(s)	No commercially available Eukaryotic cell lines were used in the study. Only patient-derived cell lines generated in our laboratory were used in the study as described in the methods. Murine GBM cell line was procured from laboratory of Dr. Maria Castro (original source of cells).
Authentication	Patient-derived cell lines were generated in our laboratory and extensively validated by whole genome sequencing. Cultures were monitored at regular intervals by STR analysis to ensure there is no mixing of patient samples, and we also did copy number analysis using CGH array in all cultures.
Mycoplasma contamination	Cell lines were periodically tested for mycoplasma contamination, and no contamination was observed.
Commonly misidentified lines<br>(See ICLAC register)	No misidentified cell lines were used in the study

Animals and other research organisms

Policy information about studies involving animals; ARRIVE guidelines recommended for reporting animal research, and Sex and Gender in Research

Laboratory animals	Strains: NOD SCID Gamma and C57BL6 mice were used at 8-12 weeks of age.
Wild animals	No wild animals were used in the study
Reporting on sex	Both sexes were included in all animal expriments, and we did not observe any sex-specific differences in effects
Field-collected samples	Study does not involve Field-collected samples
Ethics oversight	All animal studies were performed according to approved protocols by the institutional animal care and use committee at UCLA. NSG mice were housed in isolation cages with a maximum of 5 mice per cage in a separate germ-free colony with a 12 hour light/dark cycle at temperature of 20-26c with humidity 30-70% by the division of laboratory and animal medicine (DLAM), UCLA. C57BL6 were housed in a separate room with 5 mice per cage with a 12 hour light/dark cycle, temperature of 20-26C and humidity maintained between 30-70% with enough nesting material and food. Animal survival experiments were assessed as follows according to Institutional protocols. “Survival” is intended to mean the time at which animals become symptomatic via the criteria of lethargy, decreased activity, dehydration (decreased skin turgor and weight loss>10%), significant skin or fur changes, or interference with any bodily function, at which point the animals were be euthanized.

Note that full information on the approval of the study protocol must also be provided in the manuscript.

Flow Cytometry

Plots

Confirm that:

- [x] The axis labels state the marker and fluorochrome used (e.g. CD4-FITC).
- [x] The axis scales are clearly visible. Include numbers along axes only for bottom left plot of group (a 'group' is an analysis of identical markers).
- [x] All plots are contour plots with outliers or pseudocolor plots.
- [x] A numerical value for number of cells or percentage (with statistics) is provided.

Methodology

Sample preparation	For single cell sequencing of tumor cells from in xenografts, tumor tissue was harvested by enzymatic dissociation, followed by FACS sorting for GFP and DAPI to isolate only viable tumor cells. For in vitro experiments measuring mCherry reporter expression in gliomaspheres, cells were dissciated with Trypsin and labeled with DAPI to exclude dead cells. For examination of markers of vascular cells, cells were iharvested and resuspended in PBS. Cells were abelled with PE or APC-conjugated antibodies and appropriate isotype control antibodies as described in the methods.
Instrument	For FACS sorting, BD FACS Aria Cell Sorter was used, and for flow cytometry, BD Fortezza Analyzer was used.
Software	Data were analyzed using FlowJo Software and BD FACS Diva 2.0
Cell population abundance	Purity of the samples after FACS sorting was determined after culturing cells, and immunostaining of appropriate markers of each cell fraction. Viability was assessed by DAPI staining to exclude dead cells.
Gating strategy	Gating strategy involved using FSC-A and SSC-A to determine the target population. Unstained sample was used as negative control to determine the samples with positive staining. An example gating strategy is shown in the source data file.

- [x] Tick this box to confirm that a figure exemplifying the gating strategy is provided in the Supplementary Information.
